# The Acute Effects of Interrupting Prolonged Sitting Time in Adults with Standing and Light-Intensity Walking on Biomarkers of Cardiometabolic Health in Adults: A Systematic Review and Meta-analysis

**DOI:** 10.1007/s40279-022-01649-4

**Published:** 2022-02-11

**Authors:** Aidan J. Buffey, Matthew P. Herring, Christina K. Langley, Alan E. Donnelly, Brian P. Carson

**Affiliations:** 1 Department of Physical Education and Sport Sciences, University of Limerick, Limerick, Ireland; 2 Department of Sport and Exercise Sciences, Manchester Metropolitan University, Manchester, UK; 3 Physical Activity for Health Research Cluster, Health Research Institute, University of Limerick, Limerick, Ireland; 4 The Football Association, St. Georges Park, UK

## Abstract

**Background:**

Increasing evidence highlights that accumulating sitting time in prolonged bouts is detrimental to cardiometabolic health.

**Objectives:**

This systematic review aimed to compare the effects of fractionating prolonged sitting with frequent short bouts of standing and light-intensity walking on cardiometabolic health markers and conduct a meta-analysis for differences in systolic blood pressure (SBP), postprandial glucose and insulin.

**Methods:**

Experimental randomised crossover trials with at least three intervention arms that assessed interrupting sitting with frequent short bouts of standing and light-intensity walking over a single day compared to a prolonged sitting condition were retrieved. These studies measured at minimum one marker of cardiometabolic health in adults > 18 years. An electronic search was completed on the 2nd of August 2021, searching PubMed and Web of Science Core Collection, Scopus, Embase, Cochrane Library and APA PsycINFO. Risk of bias was assessed using a modified Downs and Black checklist. A meta-analysis was conducted using calculated Cohen’s *d* quantifying the magnitude of difference between experimental conditions.

**Results:**

Seven studies met the inclusion criteria for the systematic review. All seven studies were included within the meta-analysis for postprandial glucose, four studies were pooled for postprandial insulin and three for SBP. Biomarkers of cardiometabolic health were discussed qualitatively if fewer than three studies measured and reported the variable. A meta-analysis of seven acute, 1-day randomised crossover trials that sampled mixed-sex adults (aged > 18 years) who were predominately overweight or participants with obesity found that standing as an interruption to prolonged sitting significantly reduced postprandial glucose (∆ = − 0.31, 95% CI − 0.60, − 0.03; *z* = − 2.15, *p* < 0.04) but had no significant effect on insulin or SBP. Light-intensity walking was shown to significantly attenuate postprandial glucose (∆ = − 0.72, 95% CI − 1.03, − 0.41; *z* = − 4.57, *p* < 0.001) and insulin (∆ = − 0.83, 95% CI − 1.18, − 0.48; *z* = − 4.66, *p* < 0.001) compared to continued sitting. When comparing light-intensity walking breaks compared to standing breaks a significant reduction in glucose (∆ = − 0.30, 95% CI − 0.52, − 0.08; *z* = -2.64, *p* < 0.009) and insulin (∆ = − 0.54, 95% CI − 0.75, − 0.33; *z* = -4.98, *p* < 0.001) was observed. Both standing and light-intensity walking showed no effect on SBP.

**Conclusions:**

Frequent short interruptions of standing significantly attenuated postprandial glucose compared to prolonged sitting; however, light-intensity walking was found to represent a superior physical activity break. The feasibility and longitudinal implications of breaking sedentary behaviour with light-intensity walking should be investigated in a free-living setting.

**Registration:**

Not available.

**Supplementary Information:**

The online version contains supplementary material available at 10.1007/s40279-022-01649-4.

## Key Points

**Table:** 

This meta-analysis of seven acute studies found intermittent short breaks of standing led to a significant reduction in postprandial glucose compared to prolonged sitting.
Light-intensity walking was found to be a superior intervention compared to standing and prolonged sitting.
The effects of breaking prolonged sitting were more pronounced in overweight individuals compared to individuals with obesity, suggesting an additional metabolic compromise in individuals with obesity.

## Introduction

Sedentary behaviour (SB) such as prolonged sitting is likely to be highly habitual and is associated with poor health outcomes [1]. SB is defined as any waking behaviour expending ≤ 1.5 metabolic equivalent tasks (METs) whilst seated, lying or in a reclined posture [2, 3]. Cross-sectional studies indicate that total time spent sedentary, independent of exercise, has been detrimentally associated with several biomarkers [4]. In individuals with known risk factors for type 2 diabetes mellitus, total sedentary time, after adjustment for moderate to vigorous physical activity (MVPA) and other covariates, was negatively associated with 2-h glucose, triacylglycerol and high-density lipoprotein (HDL) cholesterol [5]. The indication that sedentary time is independently associated with cardiometabolic health has brought a shift in research paradigms towards identifying feasible interventions to break bouts of prolonged sitting.

Any interruption to prolonged sitting can be referred to as a sedentary break [2]. A seminal study found a beneficial association between the frequency of interruptions to an individual’s sedentary time with metabolic health markers such as 2-h plasma glucose, triglycerides and measures of adiposity [6]. More recently, an average of ten additional sedentary breaks per day was shown to be beneficially associated with systolic blood pressure (SBP), HDL cholesterol, insulin, glucose, triglycerides and waist circumference [2]. The associations reported were independent of total sedentary time, MVPA [2, 6] and mean intensity of the sedentary breaks [6]. These findings support the hypothesis that the pattern in which sedentary time is accrued may be as important as total amount of sedentary time [6]. This had led to the promotion of standing as a sedentary break and growing research utilising standing interventions, as the act of standing is feasible for most individuals and environments.

Currently, the literature is unclear on the effects of brief postural changes, specifically the transition from sitting to standing and whether the transition is a sufficient stimulus to elicit metabolic and or vascular benefits [7, 8]. Previous studies have broken prolonged sitting with different modalities of physical activity (PA), largely without addressing the postural change, with researchers concluding that the extent of improvements during walking on glucose metabolism is unclear due to postural changes and increases in energy expenditure [9]. The act of standing not only achieves a change in posture, but also involves the contraction of the postural skeletal muscles and induces compensatory changes in blood pressure, heart rate and vascular tone [8, 10]. Therefore, short bouts of standing have been shown to elicit similar changes as light-intensity walking, that break prolonged sitting, which may both be more feasible than MVPA and could offer an alternative approach to improve health and is worthy of investigation [11].

### Rationale and Aims

A recent systematic review and meta-analysis compared the effects of breaking up prolonged sitting with different PA modalities in comparison to continued sitting. However, this review excluded studies that broke continued sitting with standing [12]. Two earlier systematic reviews with meta-analyses have investigated intermittent standing breaks compared to prolonged sitting [13, 14]. One review pooled two studies and showed no effect on glucose of intermittent standing compared to prolonged sitting, and were unable to investigate insulin within their meta-analysis due to data only being available from one of the included studies [13]. The second systematic review pooled five studies (glucose) and four studies (insulin) but found no effect on glucose or insulin response when comparing intermittent standing with prolonged sitting [14]. Neither of these studies compared intermittent standing breaks with light-intensity walking breaks [12–14]. The authors did state that the small number of studies may have limited the statistical power and that future research should continue to investigate the impact of intermittent standing breaks [14]. It has been suggested that standing as an intervention to break prolonged bouts of sitting may have potential physiological effects that should be explored [12]. With the increased promotion of standing desks, breaks and meetings, along with early evidence that breaking prolonged sedentary behaviour, despite the intensity of the break, can be beneficial in the improvement of cardiometabolic health markers [6]. This increased promotion of standing as a sedentary break warrants a meaningful investigation on the effects of short bouts of standing as an interruption to prolonged sitting on cardiometabolic health markers.

Therefore, the purpose of this study was to systematically review acute (1-day) experimental studies of controlled trials that compared uninterrupted sitting with conditions that fractionated prolonged sitting with frequent bouts of standing and light-intensity walking throughout a monitored day. The aim was to provide a foundation of evidence on the prescription of standing and light-intensity walking to break prolonged sitting to elicit benefits for markers of cardiometabolic health. Where data were available, a meta-analysis and meta-regression were conducted to estimate the population mean effect for standing versus sitting, walking versus sitting, and walking versus standing, and to explore sources of variability (i.e., participant and trial characteristics) in the mean effect.

### Research Questions

1. Does acutely interrupting prolonged sitting with short bouts of intermittent light-intensity walking improve cardiometabolic health markers in adults?
2. Does acutely interrupting prolonged sitting with short bouts of intermittent standing improve cardiometabolic health markers in adults?
3. Are there differences in the acute effects of interrupting prolonged sitting with short bouts of intermittent standing compared with light-intensity walking on cardiometabolic health markers in adults?

## Methods

### Data Sources and Search Strategy

This systematic review was conducted according to the PRISMA guidelines [15]. A protocol for this review was not registered prior to data extraction and therefore was not eligible for registration with such databases as PROSPERO. The PICOS framework was used a priori in the development of the search strategy:

(P) Population: mixed-sex adults (aged > 18 years), no exclusion criteria for participant characteristics or disease. (I) intervention: crossover studies with three intervention arms with a washout period between trials (1) prolonged sitting, (2) short bouts of intermittent standing, breaking prolonged sitting, and (3) short bouts of intermittent light-intensity walking, breaking prolonged sitting. (C) Comparison: studies that made three comparisons or allowed for three comparisons via the reported data (1) intermittent light-intensity walking versus prolonged sitting, (2) intermittent standing versus prolonged sitting, and (3) intermittent light-intensity walking versus intermittent standing. (O) Outcome: measured (≥ 1) biomarker of cardiometabolic health, specifically glucose, insulin, triglyceride concentrations, blood pressure (BP), heart rate, total cholesterol, HDL, non-esterified fatty acids (NEFA), flow-mediated dilation (FMD) or a measure of body composition. (S) Study design: published peer-reviewed randomised crossover trials.

Two independent investigators (AB and CL) conducted a systematic literature search, retrieving articles published prior to the 4th of January 2020, located via electronic searches of PubMed and Web of Science Core Collection databases that included eight ‘Citation Indexes’ and two ‘Chemical Indexes’ (see Electronic Supplementary Material (ESM), Point 1, for search strategy). The systematic literature search was then updated, retrieving articles published prior to the 2nd of August 2021. Additional databases included in the updated electronic searches were: Scopus, Embase, Cochrane Library, APA PsycINFO and the two initial databases PubMed and Web of Science. The reference lists of the included studies were searched manually.

### Eligibility Criteria and Paper Selection

Inclusion criteria were: (1) English language publication; (2) participants aged ≥ 18 years; (3) assessed ≥ 1 biomarker of cardiometabolic health; (4) one intervention arm consisting of prolonged sitting only; (5) assessed interrupting sitting time with (i) sitting interrupted with standing and (ii) sitting interrupted with light-intensity walking; and (6) defined periods of sitting and intensity of physical activity during breaks. All included studies had participants sit continuously except for the scheduled breaks and for use of the toilet. Studies were excluded if: (1) trials had no washout period; (2) breaks outside of the scheduled intervention breaks were not controlled; and (3) the interventions did not include standing or light-intensity walking. These criteria allowed direct comparison of standing against light-intensity walking when comparing the effects against prolonged sitting. Prolonged sitting was defined by the included studies’ durations, which varied between studies but exceeded ≥ 5 h in all included studies (see Table 1).

**Table 1:** The extracted study and participant characteristics

Study	Year	Design	Country	*n* (male/female)	Characteristics (inclusion/exclusion criteria)	Age range (years)	Mean age (years)	Height (cm)	Weight (kg)	BMI (kg/m^2^)	Waist circumference (cm)	Body fat (%)	Duration (h)
Bailey and Locke [18]	2015	RCO	UK	10 (7/3)	“Non obese” “Free from known metabolic or cardiovascular disease” “No contradictions to physical exercise”	N/A	24 ± 3	N/A	N/A	26.5 ± 4.3	N/A	N/A	5
Brocklebank et al. [10]	2017	RCO	UK	17 (8/9)	“Entirely sedentary or semi-sedentary occupation” “Chairbound with some intermittent standing” “No substantial walking or physical labour”	45–65	52.4 ± 5.1	170 ± 7.8	81.4 ± 15.1	28.0 ± 4.5	93.5 ± 10.5	N/A	5
Crespo et al. [9]	2016	RCO	USA	10 (2/8)	“Overweight or obese” “Insufficiently physically active (< 150 min of MVPA a week)”	18–55	30 ± 15	N/A	N/A	29 ± 3	N/A	N/A	8
Henson et al. [7]	2015	BIBD	UK	34 (0/34)	“Obese/overweight” “Impaired glucose regulation (Dysglycemic)” “Postmenopausal” “Excluded for regular > 150 min MVPA over a week”	50–75	66.6 ± 4.7	N/A	83.6 ± 11.7	32.9 ± 4.7	102 ± 9	N/A	7.5
Kerr et al. [8]	2017	RCO	USA	10 (0/10)	“Overweight or obese” “Postmenopausal” “Self-reported 6 h of sitting per day” “Less than 20 min MVPA on less than 3 days per week” “Impaired glucose regulation”	≥ 55	66 ± 9	161 ± 5.9	79.4 ± 12.3	30.6 ± 4.2	104.5 ± 15.2	N/A	5
Pulsford et al. [19]	2016	RCO	UK	25 (25/0)	“Weight stable” “No more than 3 PA sessions per month” “Free from known metabolic dysfunction or cardiovascular/endocrine disorders”	30–65	40.2 ± 12.2	177.3 ± 12.2	82.4 ± 17.2	26.1 ± 4.1	87.3 ± 9.4	26.6 ± 6.0	7
Yates et al. [11] *	2018	RCO	UK	60 (31/30)	“Able to walk” “Less than 75 min of self-reported vigorous exercise a week” “No glucose-lowering medication”	65–79	70 (67, 75) *	N/A	N/A	WE: 26.5 (25.0, 28.3) SA: 26.7 (23.7, 29.5)	WE: 92.0 (87.0, 98.0) SA: 95.5 (84.5, 98.0)	N/A	7.5

Data presented as mean ± SD, where (*) represents data shown as median (interquartile range)

*BIBD* balanced incomplete block design, *RCO* randomised crossover, *UK* United Kingdom, *USA* United States of America, *PA* physical activity, *MVPA* moderate-to-vigorous physical activity, *N/A* not applicable, *WE* White European, *SA* South Asian

**Fig. 1:**
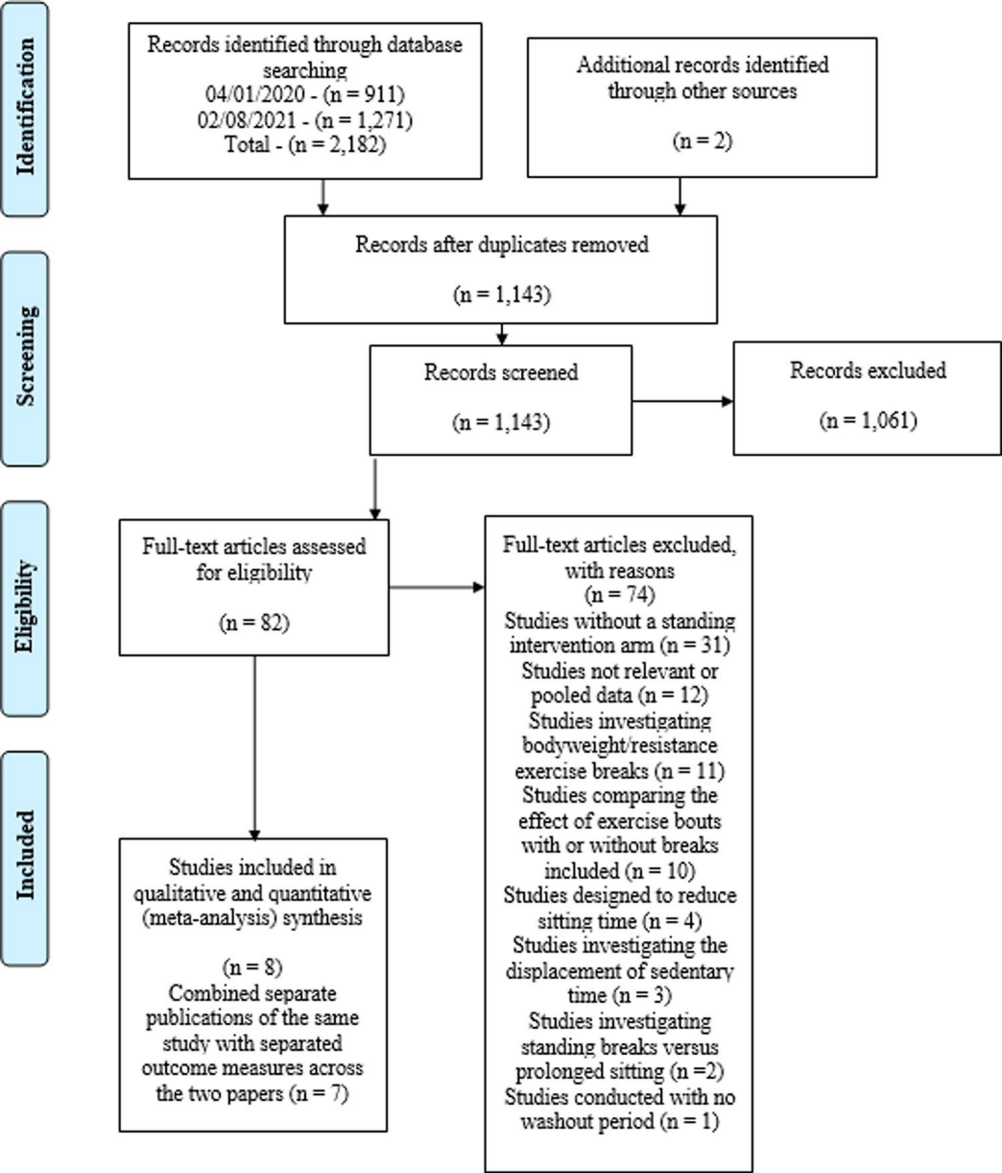
The PRISMA flow diagram illustrating the number of studies retrieved and how many were assessed for eligibility before being excluded with reasons, leaving the final *n* = 8 of included studies. Due to two of the retrieved publications being the same study that had separated outcome measures across the two papers, we decided to combine the two retrieved publications to one study leaving *n* = 7. The first date (04/01/2020) refers to the first search completed; this search strategy searched Web of Science Core Collection and PubMed databases. The second date (02/08/2021) refers to the second updated search, with the same search strategy but with the inclusion of four additional new electronic databases: Scopus, Embase, Cochrane Library and APA PsycINFO and depicts the identification, screening, eligibility assessment and final number of included studies from the retrieved articles

Retrieved articles were reviewed by two reviewers independently (AB and CL); the retrieved studies were screened firstly by title and abstract based on the inclusion criteria and then the full text. Any discrepancy regarding eligibility was discussed until consensus was reached (see Fig. 1).

### Quality Assessment

A modified version of the Downs and Black checklist was used to measure the risk of bias and quality of the included studies, evaluated independently by two reviewers (AB and CL) [16, 17]. The Downs and Black checklist measured: reporting (ten questions), external validity (three questions), internal validity (bias and confounding) (13 questions) and statistical power (one question). The modified checklist simplifies the power question and awards a single point opposed to the original five points if the study had sufficient power to detect a clinically important effect, where the probability value for a difference being due to chance is < 5%. The modified Downs and Black checklist employed had a maximum score of 28 where a score of 24–28 was considered excellent, 19–23 good, 14–18 fair, and a score < 14 poor [16]. Any disagreements between reviewers were discussed until a consensus was reached (see ESM, Point 2).

### Data Extraction and Synthesis

Microsoft Excel Sheets (Microsoft Excel, Version 2011) were developed and confirmed by the research team and used for data extraction. Participant and study characteristics were manually extracted by one author (AB) from the included studies as well as statistical data that were to assist in the meta-analysis element of the review. All descriptive information and variables reported from the included studies were extracted and included within this review (see Tables 1, 2, 3, 4). Where outcome data were not descriptively available in the included articles but presented graphically, the graph was digitised to allow for data extraction; if data extraction was not feasible the corresponding author was contacted. In instances where data were not provided, the study was excluded from the meta-analysis and discussed in the qualitative synthesis.

**Table 2:** The included studies’ intervention design, the prescription of walking (speed, environment and perceived exertion), outcome variables measured and Downs and Black checklist quality score

Study	Participants were asked to refrain from the following prior to the intervention arms for a set period	Intervention arms	Prescription of walking	Outcomes	Quality assessment (Downs and Black)
Bailey and Locke [18]	Exercise Alcohol Caffeine *For 24 h prior*	Standing: 2 min every 20 min LIPA: 2 min every 20 min	Treadmill: 3.2 km/h (Borg RPE = 6–9)	Glucose AUC Total cholesterol Triglycerides HDL Systolic BP AUC Diastolic BP AUC	Fair
Brocklebank et al. [10]	MVPA Alcohol Caffeine *For 24 h prior*	Standing: 2 min every 20 min LIPA: 2 min every 20 min	Self-perceived light intensity walking of hallways (Borg RPE = 9)	Glucose iAUC Glucose positive iAUC Glucose total AUC	Fair
Crespo et al. [9]	Exercise Alcohol Caffeine *For 24 h prior*	Standing: 10 min at 08:50 and 09:50 15 min at 10:45 and 11:45 20 min at 12:40 and 13:20 30 min at 14:00 and 15:30 LIPA: 10 min at 08:50 and 09:50 15 min at 10:45 and 11:45 20 min at 12:40 and 13:20 30 min at 14:00 and 15:30	Treadmill: 1.6 km/h	Glucose Glucose AUC Systolic BP Diastolic BP Heart rate	Fair
Henson et al. [7]	MVPA Alcohol Caffeine *For 48 h prior*	Standing: 5 min every 30 min LIPA: 5 min every 30 min	Treadmill: 1.5 to 4 km/h Average speed: 3 km/h (Borg RPE = 10–12) (Average Borg RPE = 10)	Glucose AUC Glucose iAUC Insulin iAUC NEFA iAUC Triglyceride iAUC	Fair
Kerr et al. [8]	MVPA Caffeine *For 48 h prior*	Standing 1: 2 min every 20 min Standing 2: 10 min every 60 min LIPA: 2 min every 60 min	A comfortable yet purposeful pace down hallways	Plasma glucose Glucose iAUC Plasma insulin Insulin iAUC FMD Systolic BP Diastolic BP Heart rate	Fair
Pulsford et al. [19]	Physical Activity *For 48 h prior* Alcohol Caffeine *For 24 h prior*	Standing: 2 min every 20 min LIPA: 2 min every 20 min	Treadmill: 3.2 km/h	Matsuda index Plasma glucose AUC Plasma insulin AUC SSEE	Good
Yates et al. [11]	MVPA *For 72 h prior* Alcohol *For 48 h prior*	Standing: 5 min every 30 min LIPA: 5 min every 30 min	A comfortable and of light intensity walk around a marked track within the laboratory: 2.4–4.4 km/h	Insulin AUC Glucose AUC Triglycerides AUC Systolic BP AUC	Good

*AUC* area under the curve, *Borg RPE* Borg rate of perceived exertion, *BP* blood pressure, *FMD* flow-mediated dilation, *HDL* high density lipoprotein, *iAUC* incremental area under the curve, *km/h* kilometres per hour, *LIPA* light-intensity physical activity, *SSEE* steady-state energy expenditure, *min(s)* minutes, *MVPA* moderate-to-vigorous physical activity, *NEFA* non-esterified fatty acids

**Table 3:** The baseline fasting blood measures taken prior to the intervention trials ((*) indicates that the baseline measures were taken prior to each trial, where there is not a (*) the included study only reported/measured baseline measures prior to the first trial only)

Study	Rested samples	Baseline glucose	Baseline insulin	Baseline total cholesterol	Baseline triglycerides	Baseline HOMA-IR
Bailey and Locke [18] (*)	Sat for 1 h	(mmol/L): Sit: 4.42 (4.09, 4.75) Stand: 4.32 (3.97, 4.67) Walk: 4.39 (4.04, 4.74)	N/A	(mmol/L): Sit: 4.03 (3.34, 4.73) Stand: 3.95 (3.24, 4.65) Walk: 4.11 (3.32, 4.89)	(mmol/L): Sit: 0.83 (0.77, 0.9) Stand: 0.82 (0.76, 0.87) Walk: 0.87 (0.78, 0.96)	N/A
Brocklebank et al. [10] (*)	N/A	(mmol/L): Sit: 5.2 ± 0.6 (3.9–6.1) Stand: 5.5 ± 0.6 (4.5–7.3) Walk: 5.6 ± 0.6 (4.4–6.9)	N/A	N/A	N/A	N/A
Crespo et al. [9]	N/A	N/A	N/A	N/A	N/A	N/A
Henson et al. [7]	Sat for 1 h	(mmol/L): 5.4 ± 0.4	N/A	(mmol/L): 5.60 ± 0.87	(mmol/L): 2.17 ± 0.86	N/A
Kerr et al. [8]	Sat for 1 h	(mg/dL): 107.2 ± 17.4	uIU/ml 9.3 ± 4.8	N/A	N/A	2.5 ± 1.5
Pulsford et al. [19]	08:30–10:00 Canula inserted during this time	(mmol/L): 4.3 ± 0.8	(pmol/L): 66.6 ± 33.5	(mmol/L): 4.9 ± 0.7	(mmol/L): 0.9 ± 0.5	1.4 ± 0.6
Yates et al. [11]	Canula inserted then sat for 1 h	(mmol/L): WE: 5.0 (4.4, 5.5) SA: 5.1 (4.7, 5.6)	(mU/L): WE: 6.6 (4.4, 5.5) SA: 11.0 (7.6, 13.4)	(mmol/L): WE: 4.7 (3.8, 5.6) SA: 4.4 (3.8, 5.1)	(mmol/L): WE: 0.7 (0.5, 0.9) SA: 1.1 (0.8, 1.4)	WE: 1.48 (0.97, 2.54) SA: 2.25 (1.51, 3.46)

*SA* South Asian, *WE* White European, *N/A* not applicable

**Table 4:** The meal composition prior to a trial date and if participants needed to fast and for how long, the evening before a trial. The table also illustrates the provided meals and drinks of the included studies, the timings of meals/drinks, total energy consumed and macronutrient composition of the provided meals/drinks

Study	Standardised evening meal prior to intervention arm	Were participants asked to fast and for how long	Number of meals and drinks during the trial	Energy	Carbohydrates	Fats	Protein
Bailey and Locke [18]	No	Yes: Overnight	Two drinks consumed at once (after 1 h of uninterrupted sitting)	740 kcal	80.3 g (sugars = 4.0 g)	50 g (saturated = 5.3 g)	Nil
Brocklebank et al. [10]	Yes, Two Choices: 637/610 kcal 44/50% of energy from Carbohydrates 33/36% from Fat 18/12% from Protein	Yes: Overnight	Two drinks consumed at once (at the start of the trial)	600 kcal	73.6 g	23.2 g	23.6 g
Crespo et al. [9]	Yes: Participants consumed the same dinner as test day	N/A	Three meals/drinks: Breakfast, lunch and dinner	Breakfast: 479 ± 18 kcal Lunch: 543 ± 10 kcal Dinner: 743 ± 16 kcal	Breakfast: 84 ± 5 g Lunch: 77 ± 3 g Dinner: 122 ± 6 g	Breakfast: 10 ± 1 g Lunch: 16 ± 1 g Dinner: 18 ± 2 g	Breakfast: 14 ± 1 g Lunch: 23 ± 2 g Dinner: 22 ± 4 g
Henson et al. [7]	Participants replicated their food and drink intake the day before the first trial before every subsequent trial	Yes: Overnight (10-h)	Two meals: Breakfast and lunch	N/A	Breakfast and lunch: 0.66 g per kg of body mass (≈55 g)	Breakfast and lunch: 0.66 g per kg of body mass (≈55 g)	Breakfast and lunch: 0.4 g per kg of body mass
Kerr et al. [8]	Yes 450–475 kcal each 52–56% from carbohydrates 23–25% from fat 21–24% from protein	Yes: Overnight (10-h)	Two meals: Breakfast and lunch	5 kcal per kg of body weight	Breakfast and lunch: 57% of meal (≈56 g)	Breakfast and lunch: 28% of meal (≈27 g)	Breakfast and lunch: 15% of meal
Pulsford et al. [19]	Participants replicated their food and drink intake the day before the first trial before every subsequent trial	Yes: Overnight (12.5 h)	One meal: Lunch One oral glucose tolerance test (OGTT) drink (after baseline measure)	Meal = 150 kcal per 100 ml OGTT = 1244.74 kJ	Meal: 18.4 g per 100 ml (≈92 g) OGTT: 75 g of glucose	Meal: 5.8 g per 100 ml (≈29 g)	Meal: 6.0 g per 100 ml
Yates et al. [11]	Participants replicated their food and drink intake 48 h before trial 1 for all subsequent trials	Yes: Overnight (10 h)	Two identical meals: Breakfast and Lunch	8 kcal per kg of body weight	52% of meal	35% of meal	13% of meal

*OGTT* oral glucose tolerance test

### Meta-analysis

Papers that provided unadjusted mean and standard deviation (SD) for postprandial glucose [7–11, 18, 19], insulin [7, 8, 11, 19] and SBP [8–11] for sitting, standing and walking conditions were included in meta-analysis. Cohen’s *d* was calculated to quantify the magnitude of difference in change across time between standing and sitting conditions, walking and sitting conditions, and walking and standing conditions per standard guidelines; effect sizes of 0.2, 0.5 and 0.8 were considered small, moderate and large, respectively [20]. Effect sizes were calculated such that larger improvements for standing versus sitting, walking versus sitting, and walking versus standing resulted in negative effect sizes (i.e., greater reduction in biomarkers of poor health).

Using SPSS macros (SPSS *MeanES, MetaReg*), random effects models were used to aggregate mean effect size delta (Δ) and to test variation in the effects according to potential moderators, including participant and trial characteristics [21, 22]. Heterogeneity and consistency were examined with the *Q* statistic and *I*^2^, respectively [22, 23]. Heterogeneity was indicated if *Q*_Total_ reached a significance level of *p* ≤ 0.05 [22]. Egger’s test and Begg’s rank correlation test examined publication bias [24, 25].

#### Potential Moderators

Potential moderators, or sources of variability in the overall mean effect size that are of logical, theoretical, and/or prior empirical relation to exposure and/or outcome variables, were included in meta-analysis where there were at least three effect sizes for each level of the moderator variable (i.e., at least three of the included studies measured and reported the variable). The moderating variables included within the meta-analysis were: nationality (UK vs. USA), sample sex (mixed sex sampling vs. female only sampling), sample age (20–50 years vs. > 50 years), body mass index (BMI) [overweight vs. people with obesity] and walking breaks (2-min breaks every 20 min vs. 5-min breaks every 30 min). Other moderating variables were the prescribed dose of the intervention (fixed measured speed vs. participant’s rate of perceived exertion (RPE)), the evening meal prior to the intervention day (provided fixed meal vs. replicated normal diet) and the measurement method of postprandial glucose (area under the curve (AUC) vs. incremental area under the curve (iAUC)).

When data permitted (i.e., where *k* = 3 for levels of the moderator variable), mean effects and associated 95% confidence intervals (CIs) were calculated for each level of potential moderators. When possible and appropriate (i.e., each moderator level contained at least three effect sizes), each of the moderators was coded according to contrasts among its levels and tested with univariate meta-regression analysis with maximum likelihood using a SPSS macro (*MetaReg*) [26]. Moderator analyses were not possible for SBP.

## Results

The initial search strategy identified 911 articles and the updated search strategy identified 1271 articles. Following the screening process, eight studies met the inclusion criteria (see Fig. 1). Two of the eight articles were separate publications of the same study with separated outcome measures across the papers. These two published papers by Crespo and colleagues [9] and Ziegler and associates [27] were combined within this review so that the ambulatory blood pressure reported in one article [27] was paired with the postprandial glucose data reported in the second article [9] and will only be discussed as Crespo and colleagues [9]. Therefore, seven studies were included in the final systematic review and meta-analysis.

### Narrative Synthesis of Included Studies

The seven articles reviewed were acute experimental studies with an emphasis on interrupting prolonged sitting with bouts of standing and light-intensity walking as a ‘break’ in sedentary time. Details of the study and participant characteristics of the seven included papers are presented in Tables 1 and 2. Many of the included studies screened participant exercise/PA habits and ensured participants were not physically active [7–11, 19]. Two studies used the 150-min threshold of MVPA, excluding participants who completed > 150 min of MVPA a week [7, 9], whilst one study selected participants with ‘entirely sedentary or semi sedentary occupation’ where the participants would be chair-bound with no substantial walking or physical labour [10]. A further study excluded participants who completed more than three PA sessions per month [19] and one study screened for < 75 min of self-reported vigorous exercise a week [11]. Only one study had no criteria relating to the amount of exercise the participants completed [18], ensuring only that participants had no contraindications to physical exercise; this requirement was also observed in another study [11, 18]. Two studies excluded individuals with known metabolic dysfunction or cardiovascular diseases [18, 19], with one study excluding individuals with endocrine disorders [19]. A further study excluded participants who were taking glucose lowering medication [11], whereas two studies included participants who had been screened for impaired glucose regulation [7, 8]. Five of the studies originated from the UK, whilst the remaining two were conducted in the USA [8, 9]. Four studies recruited a mixed-sex population [9–11, 18], whilst two recruited females only [7, 8], and one recruited a male-only population [19].

### Quality Assessment and Risk of Bias

The quality of four of the included studies was mutually agreed upon with three of the studies included requiring discussion until a consensus was met. Two papers (28.6%) were evaluated as ‘good’ whilst five (71.4%) were determined as ‘fair’; the mean score and SD were 18.25 ± 1.62 (see Table 2).

The risk of bias (internal validity—confounding bias) within the included studies was low or unclear due to the included studies utilising randomised crossover study designs. The included studies minimised internal bias by conducting well-controlled trials with constant monitoring and washout periods made possible by the acute experimental nature of the trials. The individual scoring of each included study can be found in the ESM. The ‘reporting’ of the included papers scored high, with the main downfall of the included papers being failing to report adverse events consequential to the intervention [17]. While these studies may not have had participants who experienced adverse events, reporting this in the paper would still have been beneficial. The external validity of the included studies was generally low, due to the nature of the laboratory setting and the artificially induced experimental conditions. One study conducted its research within the participants’ workplace environment, increasing external validity [10]. However, internal validity was compromised due to a reduced washout period of 1 day compared to 7 days to limit the number of insertions of glucose-monitoring systems [10]. Two studies simulated an office environment within the laboratory setting [9, 19]. However, all studies restricted normal daily free-living activities and diets to prioritise internal validity. When judging the internal validity, the included studies took a varied approach to the data collection and analysis of their outcome measures. Three studies used external investigators and statisticians, thereby increasing the studies’ internal validity as they were blinded to the experimental condition, when measuring the outcome variables and performing statistical analysis [7, 8, 11].

### Study Design Protocols

There was significant heterogeneity in the design of the included studies, specifically in terms of: (i) the frequency of breaks, ranging from every 20 min to every hour [8]; (ii) the duration of breaks, ranging from 2 min [8, 10, 18, 19] to 30 min [9]; and (iii) the total time of sitting interrupted, ranging from 28 min to 2 h and 50 min (see Table 2).

When prescribing light-intensity walking breaks the included studies either utilised motorised treadmills [7–9, 9–19]; hallways [8, 10] or a marked track [11]. The definition of light-intensity varied between the studies with some studies using the Borg RPE scale [7, 10, 18], a fixed walking speed [9, 19] or a self-selected purposeful comfortable pace [8] (see Table 2). However, prescribing a fixed speed does not allow for intra-individual differences in fitness, thereby lowering external validity. The included studies that prescribed an RPE range or prescribed a self-selected pace can be considered weaker in design as they have limited control and standardisation over the intervention.

#### Difference in Meals Administered

When controlling and prescribing the food and drink consumed prior to and during the trials, the included studies took varied approaches (see Table 4). In terms of standardising the evening before the intervention arm trial, only one study did not standardise the evening meal [18], whereas two of the included studies [7, 19] had participants replicate their food and drink intake the day before their first trial for every subsequent trial and one study asked the same of their participants but to replicate their food and drink intake for the 48 h before a subsequent trial [11]. These three studies thereby did not know the macronutrient composition of the meals the day prior to the trials but had attempted to standardise across the trials. Two studies prescribed meals for the days prior [8, 10], with one study [9] prescribing the same meal on both the test day and the day prior, thereby repeating the same calories and macronutrient composition [8–10]. All but one study [9], which did not report this information in its methods section, asked their participants to fast ‘overnight’. One study simply stated ‘overnight’ [10] with no timeframe provided, whereas timeframes were provided by the other included studies such as 10 h [7, 8, 11] or 12.5 h [19]. In terms of the meals or drinks on the intervention day, two studies administered two drinks at once in the morning [10, 18], one providing carbohydrates (80.3 g), fat (50 g) and no protein [18]. The other provided a mixed macronutrient test drink with carbohydrate (73.6 g), fat (23.2 g) and protein (23.6 g) [10]. Two studies provided two identical meals to their participants (breakfast and lunch); the meals’ total energy (kcal) was based on participants’ body weight but varied with respect to the energy kg^−1^ of body mass, where one study provided 5 kcal kg^−1^ [8] and the other 8 kcal kg^−1^ of body mass [11]. However, both provided a mixed meal of macronutrients (see Table 4) [8, 11]. Only one of the included studies performed an oral glucose tolerance test, following a baseline blood sample of 75 g of glucose (1244.74 kJ) followed by a mixed macronutrient test meal at lunch as a meal replacement drink [19]. The final study provided three meals: breakfast, lunch and dinner, all consisting of mixed macronutrients with a high carbohydrate content [9].

#### Measurement of Glucose

Comparing the different approaches taken in the measurement of glucose, this review found that from the seven papers included, three collected blood through a cannula [7, 11, 19] and one a catheter [8] inserted upon the arrival of the participants. Two studies inserted a continuous glucose monitor (CGM), the iPro2 [9, 10]. The final study included in this review measured glucose via capillary sampling [18]. The differences in measurement techniques lead to variations in analysis and the number of postprandial time points measured. The iPro2 CGM measured interstitial glucose concentrations every 5 min throughout the trials, whereas the studies that sampled via cannulas or catheters recorded glucose at 30, 60, 120 and 180 min postprandial [7, 11]. One of the included studies sampled glucose more frequently but over a shorter period, measuring at baseline 30, 60, 90 and 120 min postprandial [19], whereas one study sampled blood glucose hourly from baseline at 60, 120, 180, 240 and 300 min postprandial via capillary sampling [18].

### Outcome Measures

#### Diastolic Blood Pressure

Four studies compared interrupting prolonged sitting with standing and light-intensity walking on DBP [8, 9, 11, 18]. DBP was not included in the meta-analysis as only two studies provided data, while the others provided data graphically or as a non-significant effect [8, 11]. Three studies reported that neither intermittent standing nor light-intensity walking influenced DBP [9, 11, 18]. One study showed no effect of intermittent standing compared to prolonged sitting; however, light-intensity walking significantly increased DBP [8].

#### Triglycerides, Total Cholesterol, High Density Lipoprotein and Non-Esterified Fatty Acids

Three studies measured the impact of interrupting prolonged sitting with standing and light-intensity walking on triglyceride levels, two measured postprandial triglycerides [7, 11] and one pre- and post-triglycerides [18]. Due to the difference in measurement and subsequent analysis, triglycerides were not included in the meta-analysis. Two studies reported no significant effect when interrupting prolonged sitting with either standing or light-intensity walking [7, 18]. In contrast, one study reported a significant increase in triglycerides when prolonged sitting was compared to intermittent standing, but reported no effect when comparing light-intensity walking breaks with prolonged sitting [11]. One study measured total cholesterol and HDL when performing intermittent standing and light-intensity walking breaks compared to prolonged sitting and found no significant effect [18]. One study found that both intermittent standing and light-intensity walking significantly reduced the suppression of NEFA compared to prolonged sitting with no differences between standing and light-intensity walking [7].

#### Heart Rate

Two studies investigated the effect of interrupting standing and light-intensity walking on participants’ heart rate, one study [8] showed no effect when comparing the PA breaks compared to prolonged sitting. In contrast, the second study found heart rate to significantly increase during light-intensity walking compared to prolonged sitting and intermittent standing [9].

#### Flow-Mediated Dilation

One study assessed FMD [8]. It investigated two standing conditions and reported standing for 10 min every hour significantly improved FMD whereas 2 min standing every 20 min showed no effect. Similarly, 2 min of walking every hour showed no effect after Bonferroni correction [8].

### Meta-analysis Results

All seven studies were included within the meta-analysis; however, outcome variables such as diastolic blood pressure (DBP), triglycerides, heart rate, total cholesterol, HDL, NEFA and FMD were not meta-analysed due to fewer than three studies measuring these outcome variables. Variables that were included within the meta-analysis were: postprandial glucose, insulin and SBP.

#### Glucose

**Fig. 2:**
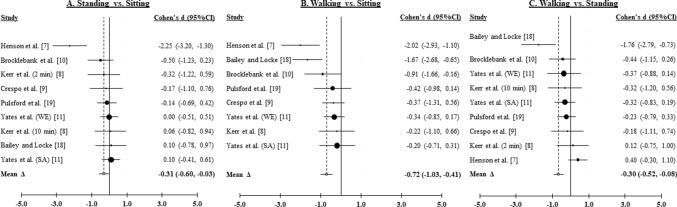
Forest plots from the postprandial glucose meta-analysis. **A** Intermittent standing breaks compared to prolonged sitting (glucose). **B** Intermittent walking breaks compared to prolonged sitting (glucose). **C** Intermittent walking breaks compared to intermittent standing breaks (glucose). *WE* White European, *SA* South Asian, *min* minutes

Nine effects were derived for standing versus sitting and walking versus standing, and eight effects were derived for walking versus sitting from seven studies [7–11, 18, 19]. Due to each study completing at minimum three trials and one study completing four trials, the total number of participants included within the glucose meta-analysis was 461. Figure 2A–C illustrate the weighted distribution of effects for glucose for standing versus sitting, walking versus sitting, and walking versus standing comparisons, respectively.

##### Standing Versus Sitting

Compared to sitting, standing resulted in a small, statistically significant mean improvement in glucose (∆ = − 0.31, 95% CI − 0.60, − 0.03; *z* = − 2.15, *p* < 0.04). The effect was heterogeneous (*Q*_8_ = 105.21, *p* < 0.001; *I*^2^ = 93.4; 95% CI 91.5, 94.8). Neither Begg’s rank correlation (Kendall *τ* = − 0.48, *p* > 0.07) nor Egger’s regression (intercept = 1.27, SE = 1.24, *p* ≥ 0.34) suggested publication bias.

Variation in the overall effect was not explained by nationality (*β* = 0.19, *p* > 0.54), participant sex (*β* = − 0.48, *p* > 0.11), participant age (*β* = − 0.26, *p* > 0.44), participant BMI status (*β* = − 0.49, *p* > 0.08), prescribed standing breaks (*β* = − 0.30, *p* > 0.40), the nature of the participants’ evening meal (*β* = − 0.21, *p* > 0.54), energy composition of the participant meal (*β* = − 0.20, *p* > 0.53), or glucose quantification method (*β* = − 0.52, *p* > 0.05) (see Table 5).

**Table 5:** Summary of univariate moderator analysis for standing versus sitting on postprandial glucose and insulin

Effect moderator	Glucose					Insulin				
	Contrast weights	Effects (k)	Δ	95% CI	*P* value	Contrast weights	Effects (k)	Δ	95% CI	*P* value
***Nationality***										
UK	− 1	6	− 0.40	− 0.76, − 0.03	**0.035**					
USA	1	3	− 0.13	− 0.37, 0.11	0.28					
***Sample sex***										
Mixed-sex	− 1	5	− 0.08	− 0.27, 0.12	0.44					
Female-only	1	3	− 0.83	− 2.18, 0.52	0.23					
***Sample age***										
20–50 y	− 1	3	− 0.11	− 0.25, 0.03	0.11					
50 + y	1	5	− 0.44	− 0.93, 0.05	0.08					
***Body mass index***										
Overweight	− 1	6	− 0.09	− 0.25, 0.07	0.29	− 1	3	− 0.01	− 0.13, 0.10	0.82
Obese	1	3	− 0.83	− 2.18, 0.52	0.23	1	3	− 0.64	− 1.54, 0.27	0.17
***Standing breaks***										
2 × 20	− 1	4	− 0.22	− 0.45, 0.01	0.06					
5 × 30	1	3	− 0.65	− 1.35, 0.05	0.07					
***Evening meal***										
Provided meal	− 1	4	− 0.26	− 0.51, − 0.002	**0.05**					
Replicated normal diet	1	4	− 0.49	− 0.96, − 0.02	**0.042**					
***Energy composition***										
Fixed calorie intake	− 1	4	− 0.20	− 0.43, 0.03	0.10					
Percentage of body weight	1	5	− 0.44	− 0.93, 0.05	0.08					
***Measurement method***										
Area under the curve	− 1	5	− 0.01	− 0.11, 0.10	0.90	− 1	3	− 0.01	− 0.13, 0.10	0.82
Incremental area under the curve	1	4	− 0.74	− 1.60, 0.11	0.09	1	3	− 0.64	− 1.54, 0.27	0.17
*P* ≤ 0.05 bolded										

##### Walking Versus Sitting

Compared to sitting, walking resulted in a moderate, statistically significant mean improvement in glucose (∆ = − 0.72, 95% CI − 1.03, − 0.41; *z* = − 4.57, *p* < 0.001). The effect was heterogeneous (*Q*_7_ = 99.39, *p* < 0.001; *I*^2^ = 94.0, 95% CI 92.2, 95.3). Begg’s rank correlation (Kendall *τ* = − 0.59, *p* < 0.05) and Egger’s regression (intercept = − 3.97, SE = 1.18, *p* ≤ 0.015) suggested possible publication bias.

Variation in the overall effect was not explained by participant age (*β* = 0.09, *p* > 0.81), prescribed walking breaks (*β* = 0.11, *p* > 0.77), the nature of the participants’ evening meal (*β* = − 0.16, *p* > 0.66), energy composition of the participants’ meal (*β* = 0.12, *p* > 0.73), or the glucose quantification method (*β* = − 0.35, *p* > 0.26).

##### Walking Versus Standing

Compared to standing, walking resulted in a small, statistically significant improvement in glucose (∆ = − 0.30, 95% CI − 0.52, − 0.08; *z* = − 2.64, *p* < 0.009). The effect was heterogeneous (*Q*_8_ = 65.14, *p* < 0.001; *I*^2^ = 89.3, 95% CI 85.8, 91.9) (see Table 6). Begg’s rank correlation (Kendall *τ* = 0.03, *p* > 0.91) was not statistically significant, but Egger’s regression (intercept = − 5.62, SE = 1.61, *p* ≤ 0.01) suggested possible publication bias.

**Table 6:** Summary of univariate moderator analysis for light-intensity versus sitting on postprandial glucose

Effect moderator	Glucose				
	Contrast weights	Effects (k)	Δ	95% CI	*P* value
***Sample age***					
20–50 y	− 1	3	− 0.79	− 1.46, − 0.11	**0.023**
50 + y	1	4	− 0.65	− 1.13, − 0.18	**0.008**
***Walking breaks***					
2 × 20	− 1	3	− 0.95	− 1.55, − 0.35	**0.003**
5 × 30	1	3	− 0.79	− 1.37, − 0.21	**0.008**
***Prescribed dose/intervention***					
Fixed speed	− 1	4	− 1.10	− 1.93, − 0.27	**0.009**
Participant’s RPE	1	4	− 0.40	− 0.66, − 0.15	**0.002**
***Evening meal***					
Provided meal	− 1	3	− 0.52	− 0.98, − 0.06	**0.03**
Replicated normal diet	1	4	− 0.67	− 1.06, − 0.28	** < 0.001**
***Energy composition***					
Fixed calorie intake	− 1	4	− 0.81	− 1.28, − 0.34	** < 0.001**
Percentage of body weight	1	4	− 0.65	− 1.13, − 0.18	**0.008**
***Measurement method***					
Area under the curve	− 1	5	− 0.49	− 0.73, − 0.24	** < 0.001**
Incremental area under the curve	1	3	− 1.04	− 1.97, − 0.13	**0.03**
*P* ≤ 0.05 bolded					

Participant sex (*β* = 0.56) and BMI status (*β* = 0.53) were significantly associated with the overall mean effect of walking compared to standing on glucose. Significantly larger improvements in glucose were derived from studies of mixed samples of males and females (∆ = − 0.52) compared to studies of females only (∆ = 0.09; *z* = 2.08, *p* < 0.04), and from studies of participants who were classified as overweight (∆ = − 0.45) compared to studies of participants with obesity (∆ = 0.09, *z* = 2.06, *p* < 0.04). Variation in the overall effect was not explained by nationality (*β* = 0.24, *p* > 0.42), participant age (*β* = 0.48, *p* > 0.10), prescribed standing breaks (*β* = 0.36, *p* > 0.28), prescribed walking breaks (*β* = 0.52, *p* > 0.11), the nature of the participants’ evening meal (*β* = 0.13, *p* > 0.70), energy composition of the participant meal (*β* = 0.47, *p* > 0.09), or the glucose quantification method (*β* = 0.44, *p* > 0.12) (see Table 7).

**Table 7:** Summary of univariate moderator analysis for light-intensity versus standing on postprandial glucose and insulin

Effect moderator	Glucose					Insulin					
	Contrast weights	Effects (k)	Δ	95% CI	*P* value	Contrast weights	Effects (k)	Δ	95% CI	*P* value	
***Nationality***											
UK	− 1	6	− 0.37	− 0.65, − 0.10	**0.008**						
USA	1	3	− 0.12	− 0.39, 0.14	0.36						
***Sample sex***											
Mixed-sex	− 1	5	− 0.52	− 0.78, − 0.26	** < 0.001**						
Female-only	1	3	0.09	− 0.34, 0.51	0.69						
***Sample age***											
20–50 y	− 1	3	− 0.69	− 1.50, 0.11	0.09						
50 + y	1	5	− 0.11	− 0.39, 0.17	0.43						
***Body mass index***											
Overweight	− 1	6	− 0.45	− 0.65, − 0.24	** < 0.001**	− 1	3	− 0.53	− 0.67, − 0.39	** < 0.001**	
Obese	1	3	0.09	− 0.34, 0.51	0.69	1	3	− 0.51	− 1.24, 0.22	0.17	
***Standing breaks***											
2 × 20	− 1	4	− 0.53	− 1.02, − 0.04	**0.04**						
5 × 30	1	3	− 0.11	− 0.47, 0.25	0.54						
***Walking breaks***											
2 × 20	− 1	3	− 0.74	− 1.34, − 0.15	**0.015**						
5 × 30	1	3	− 0.11	− 0.47, 0.25	0.54						
***Prescribed dose/intervention***											
Fixed speed	− 1	4	− 0.40	− 1.03, 0.22	0.21						
Participant’s RPE	1	4	− 0.32	− 0.45, − 0.18	** < 0.001**						
***Evening meal***											
Provided meal	− 1	4	− 0.23	− 0.49, 0.02	0.08						
Replicated normal diet	1	4	− 0.15	− 0.40, 0.10	0.24						
***Energy composition***											
Fixed calorie intake	− 1	4	− 0.60	− 1.07, − 0.13	**0.012**						
Percentage of body weight	1	5	− 0.11	− 0.39, 0.17	0.43						
***Measurement method***											
Area under the curve	− 1	5	− 0.46	− 0.70, − 0.21	** < 0.001**	− 1	3	− 0.53	− 0.67, − 0.39	** < 0.001**	
Incremental area under the curve	1	4	− 0.06	− 0.50, 0.38	0.80	1	3	− 0.51	− 1.24, 0.22	0.17	

*P* ≤ 0.05 bolded

#### Postprandial Insulin

**Fig. 3:**
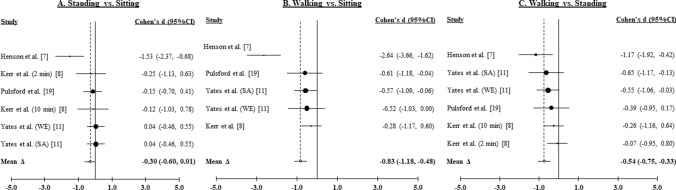
Forest plots from the postprandial insulin meta-analysis. (**A**) Intermittent standing breaks compared to prolonged sitting (insulin). (**B**) Intermittent walking breaks compared to prolonged siting (insulin). (**C**) Intermittent walking breaks compared to intermittent standing breaks (insulin). *WE* White European, *SA* South Asian, *min* minutes

Six effects were derived for standing versus sitting and walking versus standing, and five effects were derived for walking versus sitting, from four studies [7, 8, 11, 19] of 358 participants. Figure 3A–C illustrate the weighted distribution of effects for insulin for standing versus sitting, walking versus sitting, and walking versus standing comparisons, respectively.

##### Standing Versus Sitting

Compared to sitting, standing resulted in a small, non-significant improvement in insulin (∆ = − 0.30, 95% CI − 0.60, 0.01; *z* = − 1.89, *p* < 0.06). The effect was heterogeneous (*Q*_5_ = 67.71, *p* < 0.001; *I*^*2*^ = 94.1, 95% CI 92.0, 95.7). Neither Begg’s rank correlation (Kendall *τ* = − 0.55, *p* ≥ 0.15) nor Egger’s regression (intercept = 1.49, SE = 1.41, *p* ≥ 0.35) suggested publication bias. Variation in the overall mean effect was not explained by insulin quantification method (*β* = − 0.58, *p* > 0.06) or participant BMI status (*β* = − 0.58, *p* > 0.06) (see Table 5).

##### Walking Versus Sitting

Compared to sitting, walking resulted in a moderate, statistically significant improvement in insulin (∆ = − 0.83, 95% CI − 1.18, − 0.48; *z* = − 4.66, *p* < 0.001). The effect was heterogeneous (*Q*_4_ = 61.06, *p* < 0.001; *I*^2^ = 95.1, 95% CI 93.1, 96.5). Begg’s rank correlation (Kendall *τ* = − 0.32, *p* ≥ 0.44) was not statistically significant, but Egger’s regression (intercept = − 8.58, SE = 1.43, *p* ≤ 0.009) suggested possible publication bias.

##### Walking Versus Standing

Compared to standing, walking resulted in a moderate, statistically significant improvement in insulin (∆ = − 0.54, 95% CI − 0.75, − 0.33; *z* = − 4.98, *p* < 0.001). The effect was heterogeneous (*Q*_5_ = 31.22, *p* < 0.001; *I*^2^ = 87.2, 95% CI 81.2, 91.3). Begg’s rank correlation (Kendall *τ* = 0.28, *p* > 0.44) was not statistically significant, but Egger’s regression (intercept = − 10.21, SE = 1.93, *p* ≤ 0.006) suggested possible population bias. Variation in the overall mean effect was not explained by insulin quantification method (*β* = − 0.02, *p* > 0.95) or participant BMI status (*β* = − 0.02, *p* > 0.95) (see Table 7).

#### Systolic Blood Pressure

**Fig. 4:**
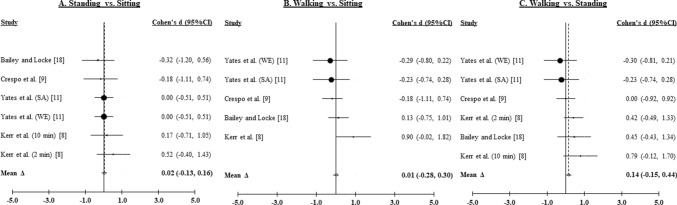
Forest plots from the postprandial systolic blood pressure (SBP) meta-analysis. **A** Intermittent standing breaks compared to prolonged sitting (SBP). **B** Intermittent walking breaks compared to prolonged siting (SBP). **C** Intermittent walking breaks compared to intermittent standing breaks (SBP). *WE* White European, *SA* South Asian, *min* minutes

Six effects were derived for standing versus sitting and walking versus standing, and five effects were derived for walking versus sitting, from four studies [9–11, 18] of 296 participants. Figure 4A–C illustrate the weighted distribution of effects for SBP for standing versus sitting, walking versus sitting, and walking versus standing comparisons, respectively.

##### Standing Versus Sitting

Compared to sitting, standing did not result in improvement in SBP (∆ = 0.02, 95% CI − 0.13, 0.16; *z* = 0.22, *p* > 0.82). The effect was not heterogeneous (*Q*_5_ = 9.56, *p* > 0.08; *I*^2^ = 58.2, 95% CI 31.4, 74.5). Neither Begg’s rank correlation (Kendall *τ* = − 0.15, *p* > 0.68) nor Egger’s regression (intercept = − 0.06, SE = 0.51, *p* > 0.91) suggested publication bias.

##### Walking Versus Sitting

Compared to sitting, walking did not change SBP (∆ = 0.01, 95% CI − 0.28, 0.30; *z* = 0.09, *p* > 0.93). The effect was heterogeneous (*Q*_4_ = 29.81, *p* < 0.001; *I*^2^ = 89.9, 95% CI 84.8, 93.3). Begg’s rank correlation (Kendall *τ* = 0.67, *p* ≥ 0.11) was not statistically significant, but Egger’s regression (intercept = − 5.48, SE = 0.78, *p* ≤ 0.006) suggested possible population bias.

##### Walking Versus Standing

Compared to standing, walking did not result in improvement in SBP (∆ = 0.14, 95% CI − 0.15, 0.44; *z* = 0.94, *p* > 0.34). The effect was heterogeneous (*Q*_5_ = 41.40, *p* < 0.001; *I*^2^ = 90.3, 95% CI 86.2, 93.2). Begg’s rank correlation (Kendall *τ* = 0.55, *p* ≥ 0.15) was not statistically significant, but Egger’s regression (intercept = − 5.74, SE = 0.61, *p* ≤ 0.001) suggested possible population bias.

## Discussion

This review and meta-analysis examined experimental studies that interrupted individuals’ sitting time in an acute laboratory setting with frequent short bouts of standing and light-intensity walking, measuring the effects on cardiometabolic health markers. The meta-analytical component of this review found both standing and light-intensity walking improve postprandial glucose metabolism compared to prolonged sitting. This is a novel finding as, previously, standing had not been shown to be beneficial as a form of PA break for glucose metabolism in earlier meta-analyses [13, 14]. However, we found light-intensity walking elicited a significantly greater attenuation in postprandial glucose compared to prolonged sitting and standing breaks, which supports the findings of previous studies [13, 14]. Light-intensity walking was also shown to significantly improve postprandial insulin compared to prolonged sitting and standing breaks, but intermittent standing bouts showed no significant effect on postprandial insulin compared to prolonged sitting within this meta-analysis. Therefore, this synthesised evidence suggests that breaking up prolonged sitting with light-intensity walking is a superior intervention to breaking sitting with periods of standing but that short bouts of standing can elicit an improvement in postprandial glucose as a sedentary break to prolonged sitting.

### Metabolic Biomarkers

The pooled effects showed a positive effect on glucose metabolism in response to a meal when standing was compared with the sitting condition. However, this effect was small and heterogeneous and was not explained by any of the included moderating variables. Due to the small number of included studies, differences in outcome variables measured, and descriptive information reported, not all possible moderating factors (< 3 effects) could be included (i.e., fasting glucose). The included studies varied the prescribed break duration and frequency, which means the total time displaced from sitting to standing differed between the studies, which may plausibly explain some of the heterogeneity. When sitting was interrupted by walking, a greater, moderate effect was observed for glucose metabolism. Furthermore, walking had a small positive effect on glucose metabolism in comparison to standing breaks. This effect was moderated by sex and BMI, with significantly larger improvements in postprandial glucose being associated with mixed-sex samples compared to studies which included females only. Regarding BMI, individuals who were classified as overweight showed significantly larger improvements in postprandial glucose when completing light-intensity walking as a sedentary break compared to individuals with obesity. This may suggest further compromised metabolism in individuals with obesity compared to overweight individuals. The two studies that sampled female only participants also had the highest mean BMI (see Table 1) of the included studies falling within the obesity range [7, 8]. One study sampled postmenopausal women who were screened as dysglycemic [7] and the second study included participants with impaired glucose regulation and signs of insulin resistance [8]. These two included studies sampled participants with the highest recorded fasting glucose values, BMI and waist circumference measurements compared to the other included studies that provided this descriptive information [7, 8]. Previously insulin sensitivity has been shown to differ by sex [28]. Typically, females exhibit lower skeletal muscle mass and increased adipose tissue, which may contribute to an increase in insulin resistance [28]. Insulin resistance has previously been associated with BMI at any grade of weight gain; however, differences in body fat distribution can cause variations in insulin sensitivity [29]. BMI estimates general adiposity, however, and does not differentiate between peripheral or central adiposity, which has been shown to be a contributing risk factor to insulin resistance [29, 30]. Due to the small number of studies and the descriptive variables provided, we were unable to include waist circumference as a moderating variable, which may have provided a better indication of central adiposity and visceral adipose tissue [30]. Therefore, we speculate that these effects may be influenced by impaired metabolism related to poor body composition in these female-only studies involving participants who present with obesity, resulting in less favourable positive metabolic benefits when performing intermittent light-intensity walking.

These findings suggest that standing breaks have a small beneficial effect compared to prolonged sitting on glucose metabolism, but walking breaks represent a superior intervention. The mean reduction of postprandial glucose across the seven included studies when completing intermittent standing breaks compared to prolonged sitting was − 9.51% ± 13.95 (ranging from a − 33.96% decrease to a 4.29% increase), whereas light-intensity walking was shown to reduce postprandial glucose by − 17.01% ± 15.42 (ranging from − 55.64 to − 3.28%) when compared to prolonged sitting. This is unsurprising; however, this meta-analysis is the first to report a statistically significant mean improvement in postprandial glucose response when interrupting prolonged sitting with standing. Two previous meta-analyses investigated standing as a sedentary break compared to prolonged sitting and found no significant difference in postprandial glucose [13, 14]. Both previous meta-analyses suggested that standing may not be a sufficient stimulus in the improvement of postprandial glucose [13, 14]. However, Saunders and colleagues suggested that due to the small number of papers (five) included in their review that implemented standing breaks, future research should continue to investigate standing as a sedentary break [14]. The second earlier review only included two studies within their meta-analysis when investigating postprandial glucose [13]. Within this current systematic review, we retrieved and included a greater number of studies (seven), allowing nine effects to be derived, and included a study that recruited 60 participants who had not been included in the two previous reviews, greatly increasing the sample size [11, 13, 14]. Within this current review we also included two studies that sampled participants with impaired glucose regulation, one of which was included in a previous systematic review but not both [14] or either in the earliest systematic review [13]. This may account for the difference in findings between this current systematic review and the previous two, which investigated the effects of standing breaks compared to prolonged sitting on postprandial glucose [13, 14].

Walking was investigated recently in a large meta-analysis that compared interrupting prolonged sitting with a multitude of intermittent physical ‘activities’ [12]. This meta-analysis excluded studies that interrupted prolonged sitting with standing; however, they grouped light to moderate intensity walking, jogging and cycling within their analysis [12]. They reported moderate effect sizes (standardized mean difference (SMD)) and statistically significant reductions in glucose (SMD =  − 0.54, 95% CI − 0.70, − 0.37, *p* < 0.001) when compared to prolonged sitting. These findings are similar to those presented here, although with smaller effect sizes.

Standing as a sedentary break showed no significant effect on postprandial insulin compared to prolonged sitting (*p* < 0.06). The number of included studies that measured postprandial insulin (*n* = 4) was lower compared to the number that measured postprandial glucose (*n* = 7), and perhaps with an increased number of studies or larger sample size we may have seen an effect on postprandial insulin. Light-intensity walking showed a moderate, statistically significant improvement when compared to continued sitting and standing breaks on postprandial insulin. This reiterates the previous finding related to glucose metabolism, that light-intensity walking provides a superior stimulus compared to interrupting prolonged sitting with standing, which fails to elicit an attenuation in postprandial insulin. Taken together, an attenuation in both postprandial glucose and insulin is suggestive of an increase in insulin sensitivity and decreased insulin secretion, which has been associated with preservation of pancreatic beta-cell function [31]. This finding strengthens previous research that has shown breaking prolonged sitting with light to moderate PA reduces postprandial insulin concentrations [12–14]. Previous reviews that included a meta-analysis relating to insulin have either excluded studies that utilised standing as a sedentary break [12] or were unable to include standing within the meta-analysis due to the number of studies retrieved [13]. One previous meta-analysis found no effect of standing breaks when compared to prolonged sitting, and they noted that short durations (< 10 min) of standing as an interruption to sitting may not be capable of inducing a reduction in insulin response [14]. Our findings agree with this conclusion, suggesting the low-intensity nature of standing is not capable of preventing the detrimental effects of prolonged sitting when administered in an acute setting.

### Cardiovascular Health

Standing and light-intensity walking both showed no significant difference in SBP when compared to prolonged sitting and there was no difference in SBP between interventions. There were insufficient analyses to perform a meta-analysis on the effects of fractionating prolonged sitting on DBP. The majority of studies included showed no effect of either standing or light-intensity walking on DBP, with one study finding an elevated DBP with light-intensity walking. In total, this suggests that fractionating prolonged sitting with standing or light-intensity walking does not positively impact DBP. Currently, few studies have investigated the effects of sedentary breaks and measured the implications on blood pressure, resulting in two previous reviews excluding blood pressure from the meta-analysis [12, 14]. Similarly, only one study reported on FMD, with positive impacts observed for both standing and light-intensity walking. The included studies only inform us of the acute effect of sedentary breaks on vascular function and identify a need for a longitudinal intervention.

Three studies measured postprandial triglyceride response, none observing a reduction in triglyceride response, potentially due to a time-delayed response to the effects of PA breaks [32]. A delay of 8–16 h has been reported before the peak of lipoprotein lipase activity and the attenuation of postprandial triglyceride responses from the onset of activity [32, 33]. However, reductions in postprandial triglyceride response (2.23 mmol/L (iAUC)) compared to a prolonged sitting condition have been observed in an acute study that utilised higher intensity sedentary breaks of 2 min 32 s every hour over 8 h in sedentary adults [34]. Overall, the low intensity of the breaks and the acute setting in which postprandial triglycerides were measured within the included studies may be too short a window for observable effects to be recorded. There were too few studies (< 3) that measured total cholesterol, HDL and NEFA when performing intermittent standing and light-intensity walking breaks compared to prolonged sitting to synthetise within this review.

### Implications and Future Directions

The findings of this systematic review have implications for the grouping of heterogeneous activities under the term ‘light-intensity physical activities’ (LIPA) [35]. Light-intensity walking was shown to significantly reduce postprandial glucose and insulin compared to prolonged sitting and equal durations of intermittent standing. It has formerly been recognised that prolonged sitting reduces the contractile activity of skeletal muscle [36]. Previously, the acute increase in glucose uptake was shown to be preferentially regulated by the contraction-mediated pathway in place of the insulin-dependent pathway in a recent study investigating interrupting sedentary time with light-intensity walking [37]. This suggests the acute benefits on postprandial glucose and insulin are more pronounced during intermittent light-intensity walking breaks than standing via the contraction-mediated pathway, due to the greater intensity and frequency of concentric and eccentric muscular activity [19, 38]. The increase in muscular contractions and increased glucose uptake via the insulin-independent pathway acutely reduce insulin secretion in the maintenance of glucose homeostasis [19].

The frequency and break duration of intermittent standing and light-intensity walking were not associated with reductions in postprandial glucose and insulin within the meta-regression. However, due to the number of studies included and a large number of the included studies prescribing 2-min breaks every 20 min or 5-min breaks every 30 min, studies that did not prescribe these break durations or frequency were not included within the meta-regression (< 3 effects). It therefore remains unclear if the break duration and frequency are mediators in the cardiometabolic response to interrupting prolonged sitting [12]. Our findings build upon a previous meta-analysis and concur that future research should investigate the effects of sedentary break duration and frequency on cardiometabolic health during a standardised sedentary bout [12].

On average, the majority of the included studies interrupted their participants’ simulated sedentary behaviour for ~ 28 min; this can also be seen as an increase in LIPA. Previously Jefferis and colleagues [39] found that a daily increase of 30 min of LIPA was associated with a 17% attenuation in mortality following adjustment for sedentary time and MVPA. If intermittent sedentary breaks of standing or light-intensity walking were implemented in an individual’s daily life or workplace environment, individuals would be able to reduce the duration of their sedentary bouts and total sedentary time in addition to increasing daily total LIPA. This may be more feasible and translatable than asking the public to complete structured exercise sessions or the attainment of MVPA.

The pooled meta-analysis of participants who were largely sedentary and classified as overweight, according to their BMI, showed a significant improvement in postprandial glucose when fractionating prolonged sitting with intermittent standing. This is an important novel finding that supports the positive impact of standing on metabolic health. The meta-analysis also showed greater improvements in postprandial glucose and insulin when performing light-intensity walking compared to prolonged sitting and intermittent standing. This has implications for individuals attempting to achieve long-term glycaemic control and the management of postprandial spikes in blood glucose [40]. These findings suggest that breaking prolonged sitting with intermittent short bouts of standing and light-intensity walking can have immediate and positive effects on postprandial glucose without pharmaceutical aid [40]. This identifies a need for longitudinal studies that focus on interrupting prolonged sitting in a free-living setting over an extended period of time to test the feasibility and efficacy of standing and light-intensity walking sedentary breaks.

These findings can help to inform public health policies that will hopefully recommend sedentary breaks of light-intensity walking alongside MVPA to interrupt prolonged sitting. The conclusions of this study are confined to an acute setting. The inclusion of sedentary breaks of light-intensity walking are more viable in comparison to MVPA in working environments and for individuals with contraindications to MVPA. Whilst this review has not investigated MVPA as a sedentary break, previous reviews have, and have found beneficial effects on postprandial glucose and insulin compared to prolonged sitting [13, 14], and therefore must also be recommended when advocating the promotion of sedentary breaks. Additionally, there is some support from the findings for an attenuation of postprandial glucose following standing breaks. However, further research data may be required to strengthen the evidence base for standing breaks.

### Limitations

The quality of the included studies fell between ‘fair’ and ‘good’, with the reporting and internal validity features addressed satisfactorily, while the external validity was generally poor given the nature of the laboratory and experimental design. However, having greater internal validity was beneficial for addressing the aim of this systematic review and allowing the effects of standing to be isolated. The prolonged sitting control condition is also artificial in that in a normal free-living condition the likelihood of individuals remaining completely seated for 5–8 h concurrently, excluding comfort breaks, is relatively low. Additionally, these findings obtained in acute 1-day trials cannot easily be extrapolated to determine the long-term benefits/consequences of interrupting prolonged sedentary time [18, 31]. For transparency, though there is no consensus on the best procedure/tool to assess risk of bias, Begg’s and Egger’s tests were computed and suggested possible publication bias, and findings should be interpreted accordingly. However, available measures of bias are not very satisfactory, particularly with small sample sizes, and Egger’s test can yield a false-positive indication of bias when the overall effect is heterogeneous, as observed here [41]. The small sample of studies available and the heterogeneous measures included, as well as the heterogeneous nature of the interventions in terms of control of sedentary break time, frequency and intensity are potential confounders of the results reported.

This systematic review and meta-analysis included several outcome measures with multiple intervention comparisons. The results reported were not adjusted for multiple testing and care must be taken when interpreting the results. However, guidance surrounding multiplicity within systematic reviews are not completely satisfactory with no simple solution to the problem of multiple comparisons in systematic reviews [42]. To minimise multiple testing we determined, a priori, the outcome variables of interest and specified that meta-analyses would be performed for outcomes with three or more effects and the comparisons of interest (prolonged sitting vs. light walking breaks; prolonged sitting vs. standing breaks and light-walking breaks vs. standing breaks).

This systematic review and meta-analysis included studies that utilised a crossover research design with three arms, which allowed the direct comparison of intervention versus intervention (standing vs. light-walking breaks) and against the control condition (prolonged sitting). Due to the inclusion and exclusion criteria and the PICO framework employed in this review, studies with two arms, such as randomised controlled trials of prolonged sitting versus light-walking breaks, or prolonged sitting versus standing breaks, were excluded. Future research and reviews should investigate the effects of standing breaks compared to prolonged sitting further with the inclusion of two arm trials, due to the small number of studies available and relatively small sample sizes observed within this review.

## Conclusion

Intermittent short bouts of standing compared to prolonged sitting significantly reduced postprandial glucose in an acute 1-day setting but showed no significant effect on postprandial insulin and SBP. Light-intensity walking showed a greater attenuation of glucose and insulin compared to standing interruptions and prolonged sitting. We would, therefore, recommend light-intensity walking for clinically meaningful reductions in postprandial glucose and insulin when compared to prolonged sitting. Future research should implement sedentary breaks in a free-living setting such as the workplace environment, testing the feasibility of sedentary breaks and investigating the long-term health implications.

## Supplementary Information

Below is the link to the electronic supplementary material.

Supplementary file1 (DOCX 21 kb)

## Declarations

### Availability of data and material

The data from the systematic review and meta-analysis are presented in the article and Electronic Supplementary Material and are available from the corresponding author upon request.
