# Supplementary material for: The Acute Effects of Interrupting Prolonged Sitting Time in Adults with Standing and Light-Intensity Walking on Biomarkers of Cardiometabolic Health in Adults: A Systematic Review and Meta-analysis
Source: Sports Med. 2022 Feb 11;52(8):1765–87. doi: 10.1007/s40279-022-01649-4 (PMC9325803; doi:10.1007/s40279-022-01649-4)
Supplement: Supplementary file 1 — Supplementary file1 (DOCX 21 kb) [file 40279_2022_1649_MOESM1_ESM.docx]

**Electronic Supplementary Material Cover Page:**

Article Title: The acute effects of interrupting prolonged sitting time with standing and light-intensity walking on biomarkers of cardiometabolic health in adults: A systematic review and meta-analysis

Journal Name: Sports Medicine

Name of each author: Aidan. J. Buffey^1,3^, Matthew. P. Herring^1,3^, Christina. K. Langley^2,4^, Alan. E. Donnelly^1,3*^ and Brian. P. Carson^1,3*^

*These authors contributed equally to the research.

The affiliation(s) and names of the departments(s) and institution(s) to which the authors belong:

^1^Department of Physical Education and Sport Sciences, University of Limerick, Limerick, Ireland, ^2^Department of Sport and Exercise Sciences, Manchester Metropolitan University, Manchester, United Kingdom, ^3^Physical Activity for Health Research Cluster, Health Research Institute, University of Limerick, Limerick, Ireland, ^4^The Football Association, St. Georges Park, United Kingdom.

Address of corresponding author and email address: A. J. Buffey^1,3^,

^1^Department of Physical Education and Sport Sciences, University of Limerick, Limerick, Ireland. E-mail: [Aidan.Buffey@ul.ie](mailto:Aidan.Buffey@ul.ie). ORCID: 0000-0002-1940-1483

**Supplementary Data File:**

1. **Search Strategy:**

This search strategy was repeated on both PUBMed and Web of Science Core Databases which included eight ‘Citation Indexes’ and two ‘Chemical Indexes’:

**Web of Science Core Collection: Citation Indexes**

1. Science Citation Index Expanded (SCI-EXPANDED) --1945-present

2. Social Sciences Citation Index (SSCI) --1956-present

3. Arts & Humanities Citation Index (A&HCI) --1975-present

4. Conference Proceedings Citation Index- Science (CPCI-S) --1990-present

5. Conference Proceedings Citation Index- Social Science & Humanities (CPCI-SSH) --1990-present

6. Book Citation Index– Science (BKCI-S) --2005-present

7. Book Citation Index– Social Sciences & Humanities (BKCI-SSH) --2005-present

8. Emerging Sources Citation Index (ESCI) --2015-present

**Web of Science Core Collection: Chemical Indexes**

1. Current Chemical Reactions (CCR-EXPANDED) --1985-present (Includes Institut National de la Propriete Industrielle structure data back to 1840)

2. Index Chemicus (IC) --1993-present

**Search Strategy:**

(All Fields) (First Row): sedentary OR sitting (AND)

(All Fields) (Second Row): cardiometabolic OR cardio OR vascular OR cardiovascular (AND)

(All Fields) (Third Row): older adults OR seniors OR elderly OR geriatrics OR adults OR individuals (AND)

(All Fields) (Forth Row): break* OR interrupt* OR fraction* OR intersperse* NOT ejection fraction

1. **Downs and Black Checklist Scoring:**

Table displaying the scores for the included studies for each section of the Downs and Black Checklist and overall score.

| **Study** | **Year** | **Reporting** | **External Validity** | **Internal Validity** | **Confounding/Bias** | **Power** | **Quality Score** |
| --- | --- | --- | --- | --- | --- | --- | --- |
| Bailey and Locke [18] | 2015 | 8 | 0 | 3 | 4 | 1 | 16 – Fair |
| Brocklebank et al. [10] | 2017 | 8 | 1 | 4 | 4 | 1 | 18 – Fair |
| Crespo et al. [9] | 2016 | 7 | 0 | 4 | 4 | 0 | 15 – Fair |
| Henson et al. [7] | 2015 | 9 | 0 | 5 | 4 | 1 | 19 – Fair |
| Kerr et al. [8] | 2017 | 8 | 1 | 5 | 5 | 0 | 19 – Fair |
| Pulsford et al. [19] | 2016 | 9 | 0 | 4 | 6 | 1 | 20 – Good |
| Yates et al. [11] | 2018 | 8 | 0 | 5 | 6 | 1 | 20 - Good |
